# Caustic ingestion management: world society of emergency surgery preliminary survey of expert opinion

**DOI:** 10.1186/s13017-015-0043-4

**Published:** 2015-10-16

**Authors:** Yoram Kluger, Ofir Ben Ishay, Massimo Sartelli, Amit Katz, Luca Ansaloni, Carlos Augusto Gomez, Walter Biffl, Fausto Catena, Gustavo P. Fraga, Salomone Di Saverio, Augustin Goran, Wagih Ghnnam, Jeffry Kashuk, Ari Leppäniemi, Sanjay Marwah, Ernest E. Moore, Miklosh Bala, Damien Massalou, Chirica Mircea, Luigi Bonavina

**Affiliations:** Rambam Health Care Center, POB 9602, Haifa, 31096 Israel; Department of Surgery, Macerata Hospital, Macerata, Italy; Papa Giovanni XXIII Hospital, Bergamo, Italy; Hospital Universitário Therezinha de Jesus, Faculdade de Ciências Médicas e da Saúde de Juiz de Fora (SUPREMA), Universidade Federal de Juiz de Fora (UFJF), Minas Gerais Jiiz de Fora, Brazil; Department of Surgery, University of Florida, Gainesville, Florida USA; Parma University Hospital, Parma, Italy; Faculdade de Ciências Médicas (FCM), Unicamp Campinas, Jiiz de Fora, Brazil; Ospedale Maggiore Carlo Albert, Bologna, Italy; Department of Surgery, University Hospital Center, Zagreb, Croatia; Mansoura Faculty of Medicine, Mansoura University Egypt, Mansoura, Egypt; Assia Medical Group, Assuta Medical Centers, Tel Aviv, Israel; Meilahti Hospital, Helsinki, Finland; Department of Surgery, Post-graduate Institute of Medical Sciences, Rohtak, India; Department of Surgery, Denver Health Medical Center, Denver, USA; Hadassah - Hebrew University Medical Center, Jerusalem, Israel; Hôpital St Roch 5, Université Nice Sophia-Antipolis, Nice, France; Department of General, Endocrine and Digestive Surgery, Saint-Louis Hospital, Université Paris, Paris, France; Policlinico San Donato, University of Milan School of Medicine, Milan, Italy

## Abstract

Caustic material ingestion injuries (CMI) are uncommon. Only 5,000 cases are reported in the United States each year and most acute care healthcare facilities admit only a few cases annually. Accordingly, no single institution can claim extensive experience, and management protocols are most probably based on either expert opinion or literature reports.

In this study, we will attempt to review opinions and practices of representatives of the board members of the World Society of Emergency Surgery and compare them to the current literature.

## Introduction/Background

Caustic ingestion may result in significant injury to the entire gastrointestinal tract, but most significantly the upper tract, including the oropharyngeal cavity, larynx, esophagus, and stomach.

The majority (68 %) of cases worldwide involve children as a result of unintentional, accidental ingestion of caustic substances. The remainder of cases reported are adults with psychiatric disturbances, some after suicide attempts, or alcoholics [1, 2].

As expected, the resultant severity of injury in caustic ingestion is determined by the type of ingested substance the amount and the time of tissue exposure.

Due to the substantial morbidity and mortality associated with these injuries, the medical community demanded legislative action. Through persistent efforts, the Federal Caustic Act of 1927 was enacted, requiring appropriate labeling of caustic substances, such as lye. Subsequently, the Poison Prevention Packaging Act of 1970 directed the US Consumer Product Safety Commission to require childproof containers and improved labeling of caustics and other potentially harmful household products. These legislative acts caused dramatic decline in the occurrence of this type of injury in developed countries. However, in developing countries the incidence is still much higher [3].

While the injury pattern frequently seen in children is usually relatively minor due to smaller amounts ingested, in adults much larger quantities of the caustic substance frequently result in severe injury [3–5].

Injury caused by alkali or acid results in a different injury pattern. For example, alkali causes almost no irritation to the oral cavity, which usually results in larger ingested volumes entering the GI tract. Furthermore, because Alkali materials are thicker, they lead to longer exposure durations in the esophagus, causing progressive injury via liquefactive necrosis.

This process may take as long as two weeks to manifest itself, and is heralded by progressive thinning of the esophagus lining. The stomach and duodenum, on the other hand, are less prone to such injury, due to the neutralizing effect of gastric acids and avoidance of pyloric spasm. Accordingly, maintenance of gastric emptying results in limited exposure of the gastric mucosa to alkali.

In contrast to alkali, acids induce a burning sensation with subsequent pain immediately after contact with oral mucosa; accordingly, the volumes traditionally ingested tend to be small. In addition, since acids lack viscosity, their transit time through the esophagus is rapid.

Subsequently, the duration of exposure to the gastric mucosa is extended due to acid induced pyloric spasm, resulting in acid exposure for protracted periods of time leading to coagulative necrosis. Endogenous gastric secretion is not considered to influence this process [1, 6–8].

Acid ingestion may still cause substantial damage to the esophagus, including perforation [9].

The extent of injury that results from caustic ingestion is estimated by the depth of the resultant caustic burn. First degree burns tend to involve only the mucosa, with localized redness and edema noted at endoscopy. Second degree burns involve the mucosa and sub- mucosa with blister formation evident, while third degree burns are characterized by a transmural process that affects the entire lining with findings of extensive ulceration and necrosis appearing as gangrene [10, 11].

Of note, the clinical findings in caustic ingestion can be highly variable, and often do not correlate with the injury grade noted at endoscopy [12, 13].

**Fig. 1:**
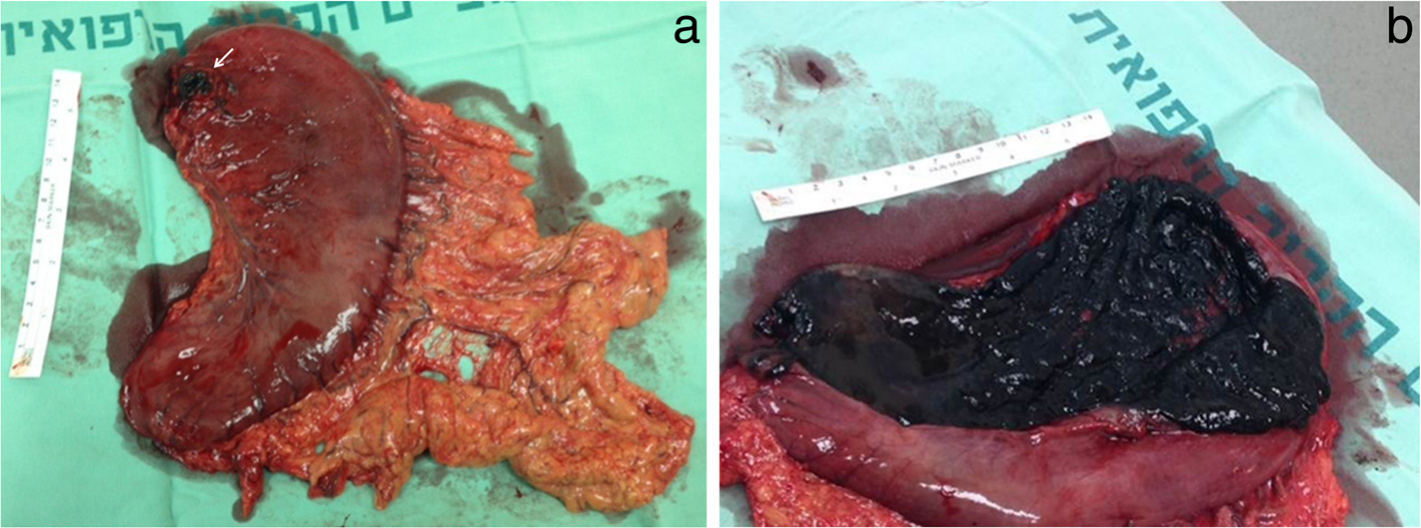
**a**: Resected stomach due to perforation (*arrow*) after caustic material ingestion. Note diffuse thrombosis of gastro-epiploic veins. **b** Stomach opened longitudinally. Note necrosis of gastric mucosa

Patients with minimal ingestion may be asymptomatic but others may experience oropharyngeal, retrosternal or epigastric pain. Findings of shortness of breath, hoarseness and stridor suggest laryngeal trauma and demand laryngoscopic evaluation. Dysphagia, odynophagia and excessive salivation are suggestive of esophageal damage, abdominal pain; vomiting and hematemesis may suggest gastric damage. Continued pain, peritonitis, tachycardia, persistent leukocytosis, acidosis and pleural effusion should raise the suspicion of perforation (Fig. 1) [14–20].

**Fig. 2:**
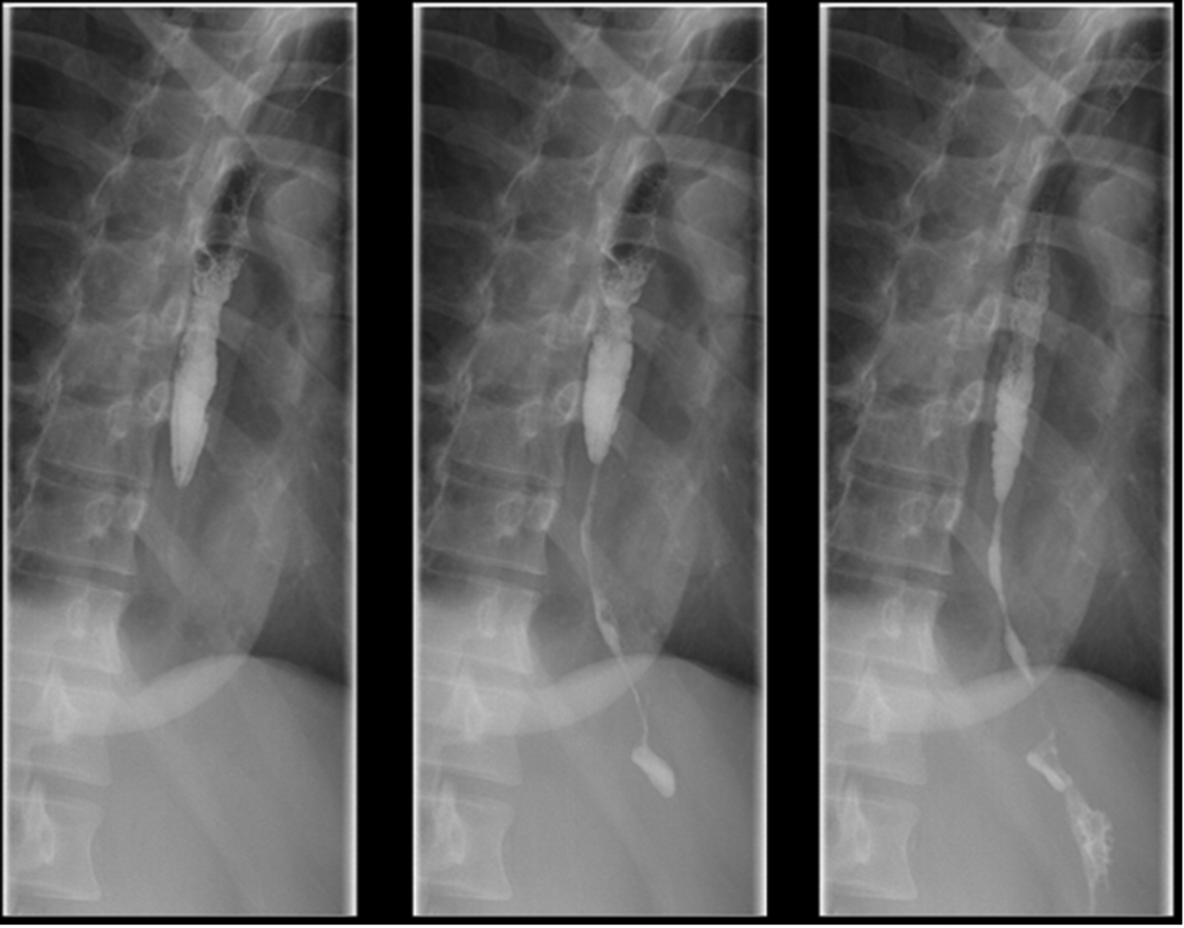
Barium swallow four month after caustic ingestion injury. Note the long stricture of distal esophagus and gastric cardia. This patient was treated with colonic interposition

Ten percent of patients sustaining CMI will experience immediate complication [21, 22]. The most common serious immediate complications after caustic material ingestion therefore include: perforation, bleeding, but late findings include fistula formation (tracheobronchial, gastro colic or even entero-aortic). Reported mortality approaches 10–20 %. Among those sustaining caustic injury in a suicide attempt, mortality may approach 75 % [6]. The delayed complications include stricture formation (Fig. 2) leading to malnutrition and long term risk of developing malignant transformation.

Although there are few published prospective studies on the management of CMI, the current review will attempt to collate all current reports and expert opinions. This work was done as a preliminary study for a consensuses conference on the topic held in Milan, Italy in March 2015.

## Method

An e-mail questionnaire was forwarded to all members of the WSES, World Society of Emergency Surgery, consisting of extensive questions pertaining to the diagnosis and treatment of caustic injuries. The question related to various aspects of diagnosis, initial management, surgical and medical treatments as well as questions on individual survey member and institutional experience with caustic ingestion injuries.

As a result of this questionnaire, a detailed literature search was performed in an effort to compare expert opinion with current available literature.

Literature search was designed in four levels and included:

1. Epidemiology, toxicology and pathophysiology of caustic injury.
2. Initial management and emergency interventions.
3. Evaluation of caustic injury – endoscopy, Computerized tomography.
4. Surgical management in the acute and late phaseof caustic injury.
5. Early and late complications.

## Results

### Survey results

#### General information

Completed survey questionnaires were obtained from the following locations world-wide: Europe 9, Asia 9, South America 4, 3 in North America, 3, and Middle East, 3.

Nineteen of the hospitals that participated in the survey reported treating 1–5 cases annually, while three facilities reported over 15 cases per year. Table 1 shows general background information about the respondents and their associated medical facilities.

**Table 1:** General information about the work environment of the respondents to the survey

Region	Europe	9	31 %
	Asia	10	34 %
	South America	4	14 %
	North America	3	10 %
	Middle East	3	10 %
Hospital size (beds)	<100	1	3 %
	101-500	9	31 %
	501-1000	10	34 %
	>1001	9	31 %
Cases of CMI encountered per year at hospital	1-5	19	66 %
	6-10	4	14 %
	11-15	3	10 %
	>15	3	10 %

#### Initial assessment and diagnostics

The preliminary treatment and imaging tests practiced by the survey participants is depicted in Table 2.

**Table 2:** Means of initial assessment and diagnostics of CMI, according to the survey respondents

Initial assessment			
Intubation	Dyspnea, stridor, edema	29	100 %
Extubation	Based on respiratory condition	25	86 %
	>7 days	4	14 %
Nasogastric tube	All patients	6	20 %
	No patients	2	7 %
	Based on endoscopy findings or evidence of oropharyngeal injury	21	72 %
	Insertion during endoscopy	20	67 %
	Insertion without endoscopy	9	33 %
	Nasogastric tube removal after >3 days	13	45 %
	Nasogastric tube removal after >7 days	16	55 %
Imaging			
Esophagography	Not performed	14	50 %
	Performed on all patients	8	29 %
	Performed only on non-intubated patients	6	21 %
Esophagogastroscopy	All patients	24	83 %
	Signs of oropharyngeal injury	3	10 %
	According to clinical development	2	7 %
	Within 12 h	19	66 %
	12-24 h	8	28 %
	Over 24 h	2	7 %
	Level of injury	17	59 %
Thoracic radiology	All patients	26	90 %
	Based on respiratory condition	3	10 %
CT	All patients	8	29 %
	Patients developing signs of peritoneal irritation or suspected perforation	20	71 %

All survey participants initiated management via oro-tracheal or naso-tracheal intubation when patients were noted to present with obvious signs of dyspnea, stridor or laryngeal edema occurred.

Of those patients who were intubated, 14 % will require ventilator support for over one week. Seventy two percent of respondents placed a nasogastric tube on initial evaluation. Twenty percent of respondents indicated that they would insert a nasogastric tube regardless of initial findings while interestingly, 6 % reported that they would avoid placement of an NG tube in these patients.

Of those placing an NG tube, 67 % suggested the importance of doing so under endoscopic guidance, 45 % reported removing the NG tube after 3 days, while the remainder (55 %) left the tube in place for over a week.

Half of survey participants were reluctant to perform esophagography, while 29 % performed the examination on all patients; 21 % performed the study only on intubated patients.

Eighty three percent performed initial esophagogastroscopy on all patients whereas the other performed the exam only according to clinical presentation or when evidence of oropharyngeal involvement was evident.

Nighty percent of survey participants performed routine chest X-ray regardless of the patient’s underlying respiratory condition. The remaining performed a CXR based upon clinical indications. Twenty nine percent of respondents routinely ordered CT scan on all patients, but the remaining group performed this test only when signs of peritoneal irritation or suspected perforation were noted.

#### Treatment

The treatment provided by survey participants is presented in Tables 3,4 and 5.

**Table 3:** The use of medical treatment for CMI, according to survey respondents

Medical treatment			
Steroids	All patients	7	25 %
	No patients	13	46 %
	Depending on the depth of injury	8	29 %
Antibiotics	All patients	10	34 %
	Only patients requiring urgent surgery	9	32 %
	Depending on the depth of injury	10	34 %

**Table 4:** The use of surgical intervention for CMI, according to survey respondents

Surgical intervention			
Surgery indication	Peritonitis, free air, peritoneal free fluid	29	100 %
	Depending on depth	3	10 %
Surgical approach	Laparotomy	15	52 %
	Possible laparoscopy	14	48 %
Reconstruction	Not perform urgent surgery	20	69 %
	If patient is stable, perform urgent surgery	9	31 %

**Table 5:** Treatment of strictures

Treatment of stricture	Endoscopy attempt if stricture is short	22	76 %
	Endoscopy attempt for short and long strictures	7	24 %

Twenty five percent of respondents administered steroids to all patients, while 29 % admistered those to patients noted to have associated second degree burns. Forty six percent of the respondents stated that they did not admister steroids in any case. Thirty four percent of respondents administered antibiotics to all patients, while 34 % suggested such a need for those patients suffering second degree burns and 32 % administered antibiotics to patients requiring surgical intervention.

All participants proceeded to urgent surgical intervention when there were signs of peritonitis, free air, or esophageal perforation a small group (3 %) said they would suggest surgical intervention in the face of extensive third degree injury. Of those proceeding to surgical intervention, 52 % performed a traditional laparotomy, while 48 % consider a laparoscopic approach. The decision to perform restorative surgery was generally based upon patient stability: 31 % of respondents attempted restorative procedures at the initial operation, while the remaining majority deferred such extensive procedures until later time frames.

Twenty four percent of respondents performed initial endoscopic treatment of short or long stricture, deferring surgical intervention for treatment failures. The majority of respondents (76 %), attempted endoscopic management of short strictures, but felt that longer strictures would require surgical intervention.

## Discussion

The diagnosis and treatment of caustic ingestion injuries has received only a modicum of attention in the literature. Furthermore, our review has identified only three randomized control trials addressing the effectiveness of steroid treatment [23–25]. Accordingly, the current relevant literature consists primarily of retrospective research and case studies.

The paucity of experience with this entity at any one center is evident from the finding that 80 % of our survey respondents treat fewer than 10 cases per year. Based on these findings, we believe that patients should be enrolled in well designed, prospective data bases; furthermore, in order to establish evidence based guidelines, a current management algorithm should be constructed based upon available knowledge.

### Diagnosis and initial treatment

The initial approach to management should involve careful assessment of the extent of injury. Hence, it is important to document the type of ingested material, quantity ingested, and an attempt to estimate the exposure duration in the various organs.

A careful assessment of symptoms is paramount. Complaints of dyspnea, dysphagia, excessive salivation, hematemesis or hoarseness suggest severe injury [27]. Although laboratory tests do not always correlate with severe injury, leukocytosis >20,000 wbc/ml, elevated CRP and pH <7.2 corroborate extent and severity of injury [1, 27–29]. Hypocalcemia may follow the ingestion of hydrogen fluoride.

Forty percent of patients suffer injury to the upper respiratory tract, and approximately 5–15 % suffers significant dyspnea, stridor or laryngeal edema, dictating immediate intubation due to imminent air way compromise [30, 31].

Initial chest X-ray may identify pneumoperitoneum, pleural effusion or pneumomediastinum. Such radiologic findings may also hint at the presence of perforation. Recent experience supports the accuracy of CT scan as a diagnostic tool with 75 % sensitivity and 90 % specificity in determining injury grade, need for surgical intervention and ability to predict complications such as stricture [32–35]. The CT injury grading system of Ryu HH et al. is based on the extent of esophageal lining edema, turbidity, paraesophageal tissue and fat hernia and presence of pleural fluid or pneumomediastinum [35].

A recent study by Lurie Y et al. demonstrated that the specificity of CT in predicting the need for operative intervention and even eventual mortality as high as was > 90 % but with sensitivity of only 30–40 %. On the basis of these facts, these authors concluded that early endoscopy may not be replaced by CT [36].

Early endoscopy (within 12–24 h following ingestion) permits careful assessment of anatomic derangements, serving as a valuable aide in decision making in order to guide the need for further interventions. Delayed endoscopy (>48 h) should be avoided due to increased risk of perforation as the resultant of tissue edema and inflammation. The grade of injury based upon careful endoscopic assessment and physical examination appears to be closely correlated with the degree of urgency for surgical intervention, the development of subsequent complications and eventual mortality [11, 37–40]. In a series by Zarger SA et al., the authors noted that all patients who succumbed to their injury had grade III burns. Furthermore, those with grade IIb and III who survived developed late complications. Lastly, with the finding of an IIa or lesser grade burn portended a complication free clinical course [11]. While III degree burns generally suggest the need for urgent surgical intervention, it should be noted that, gastrectomy or esophagectomy based on endoscopy findings alone may lead to 10–15 % unnecessary surgical procedures [33, 34, 43]. Despite these findings and the correlation of the burn depth to outcome it is interesting to note that <60 % of our respondents stated that they consistently used the injury grading assessment.

When endoscopy under anesthesia is performed by a qualified team, the risk of perforation is low and the procedure can be completed even in the presence of second or third degree burns [40–42]. It is important to avoid over-inflation of the esophagus, and also important to note that it is not always possible to pass through the burn area, and may be difficult to assess the degree of burn beyond the furthest point of view of the endoscope [1].

While it is important to maintain a high degree of suspicion during workup, is should be emphasized that upwards of 10–30 % of patients may not have damage to the esophagus or stomach; accordingly, one could argue that routine endoscopy may be unnecessary [14, 44]. In a similar way, in the pediatric population, evidence suggests that the risk of significant damage to the esophagus or stomach in those children who are asymptomatic is <2 %. Accordingly, in the pediatric group, routine endoscopy should be avoided [26, 45].

Current contraindications for esophagoscopy are obvious, overt perforation, supraglottic or epiglottic burn with edema and third degree burns in the hypopharynx [14]. Under such conditions, CT should suffice. The result of our study suggested that half the respondents were in favor of contrast studies. In the acute stage of CMI water contrast medium should be used. Barium contrast studies may be of help in evaluating stricture formation and their length in a later stage.

Recently, Endoscopic Ultrasonography (EUS) has been suggested as a helpful adjunct to evaluate patients with MCI presented for the evaluation of patients. Such an evaluation, when performed by a skilled technician, allows for excellent assessment of the degree of burn and provides for precise assessment of the depth of esophageal injury. At the current time, however, the procedure has not demonstrated an advantage in predicting immediate complications, the need of surgery and subsequent development of stricture [47–49]. Accordingly, at the current time, EUS is not being extensively used in these patients [45–50].

Regarding the use of NG tube in patients with suspected injury, our survey results suggest that 93 % inserted a nasogastric tube when evidence exists for oropharyngeal injury or when endoscopy suggests significant injury, while 7 % avoided placement of an NG tube in any scenario. Among those who supported this practice, 67 % preferred to do so while performing endoscopy. The theoretical advantage of this practice is to serve as a stent, to maintain luminal integrity, minimize stricture formation, and provide a continuous route for enteral nutrition. Of note, a number of studies have suggested that leaving the nasogastric tube for 1-2 weeks after a grade 2b or over burns reduces the need for late stricture dilatation [51–53]. Furthermore, a theoretical disadvantage, is that prolonged use of the tube could actually promote stricture formation due to fibrosis around the tube [14].

Our survey did not demonstrate any solid consensus regarding antibiotic use in this patient group. About one third of respondents indicated that they administered prophylactic antibiotics to all patients, while one third suggested using antibiotics only to those patients undergoing urgent surgery. The remaining group administered antibiotics based on the severity of the burn. Interestingly, an unproven “surgical myth” which originated in a manuscript over 60 years ago suggested that use of antibiotics in the acute phase of burn reduced stricture formation [55]. In sum, the most common current practice from our survey results appears to be administration of antibiotics only when active infection is suspected or when steroid treatment is contemplated [55–59].

There was no consensus among survey respondents regarding administration of steroids. 45 % of respondents did not administer steroids in any case, while 25 % routinely gave steroids to patients with caustic injuries. 30 % recommended administration selectively to those patients sustaining high-degree burns. Although unproven clinically, the theoretical basis for steroid administration in this group is to reduce collagen formation via alteration of fibronectin and m cytokine pathways leading to reduced stricture formation [60]. Of note, certain animal studies do suggest such an advantage, while clinical trials have failed to provide any convincing data [61–63]. One study in 1990 compared children who received prednisolone 2 mg/kg to a control group, and failed to demonstrate any reduction in stricture formation [64]. Of note, one study showed that the combined use of antibiotics, steroids and early dilation failed to reduce stricture formation and actually increased the risk of perforation [65].

This issue, however, remains far from settled. Two more recent randomized trials showed that dexamethasone (1 mg/Kg/day) as opposed to prednisolone (2 mg/Kg/day) reduced stricture formation, while another recent report showed that a combination of methylprednisolone (1 g/1.73 m^2^ per day for 3 days), Ranitidine, Ceftriaxone, and total parenteral nutrition resulted in 10–15 % stricture formation, while a group receiving the same treatment, but without methylprednisolone, resulted in upwards of 30–45 % stricture formation. In sum, all of these studies are primarily in children and suffer from limited sample size [66, 67).

Based upon the results of several meta-analyses current clinical practice suggests limiting the use of steroids to those patients with established respiratory tract edema [68, 69].

**Fig. 3:**
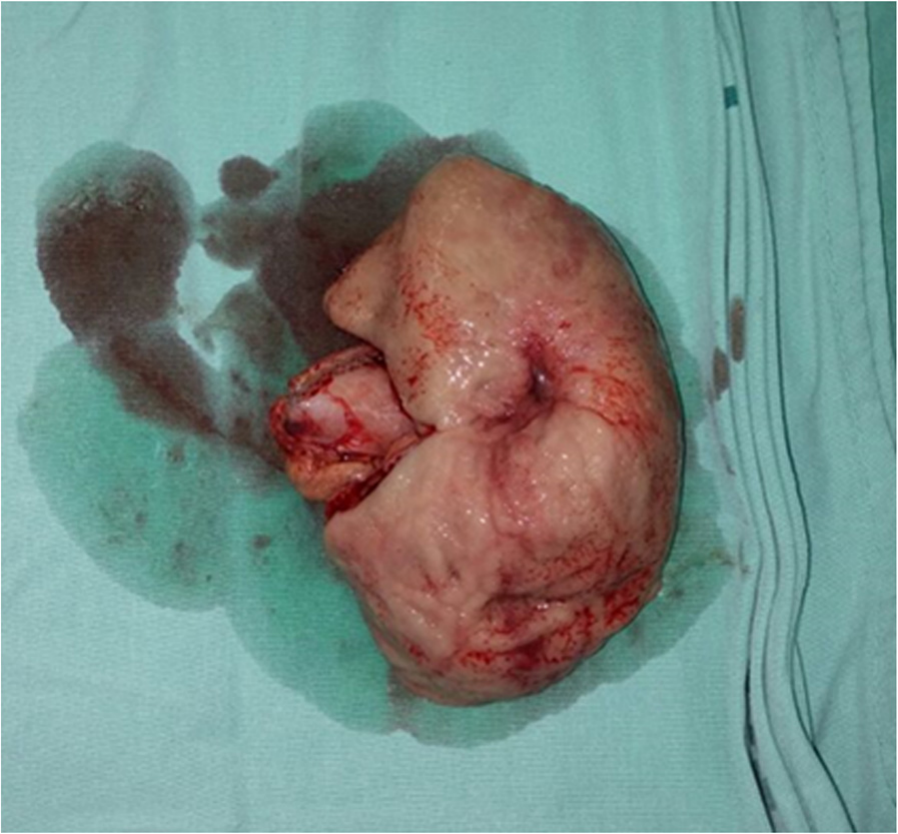
Pre-pyloric stricture explored during delayed reconstructive surgery after caustic ingestion injury

There was general agreement by all respondents that signs of peritonitis and presence of pneumoperitoneum and/or perforation of the esophagus are indications for immediate surgical intervention. Similarly, most respondents suggested that any clinical or radiological evidence of perforation also dictated urgent operation. Another late indication for urgent surgical intervention that should be recognized is bleeding due to necrosis developing several days after the initial admission [70–72]. Other abnormalities which may accompany later clinical deterioration and suggest the need for surgical intervention include: persistent acidosis, renal failure, or extensive burns requiring endoscopic evaluation [72, 73]. While most respondents performed laparotomy, initial laparoscopy was also mentioned as a viable alternative in the more stable patient, but is clearly operator dependent based upon the skill set and experience of the surgeon, as thorough exploration of the stomach and duodenum may be quite difficult for the novice laparoscopist. With sufficient skill, however, laparoscopy can be a valuable adjunct for the assessment of grade 2 or higher gastric injuries [74, 75, 77] accordingly, avoidance of gastric resection may be possible in the absence of significant damage [76]. A large esophageal perforation (rare) may require emergency esophagectomy along with cervical esophagostomy and gastrostomy whereas gastric perforation is managed with gastric resection. The need for emergency esophago- gastrectomy is rare. An additional feeding jejunostomy might prove lifesaving in such cases for the purpose enteral feeding since definitive reconstruction is possible only when injury is healed and patient is stabilized [54] (Fig. 3).

As is shown in our survey of expert opinion inconsistencies exist in regard to diagnosis and management of CIM injuries. Paradigm shifts in treatment strategies to conservative, non-operative approaches including percutaneous drainage of pleural effusions, collections or abscesses [78, 79] is perceived and indicate the need for further studies and evaluation of the current knowledge.

A further evidence based CIM management consensus initiative is indicated.
