# Erratum to: caustic ingestion management: world society of emergency surgery preliminary survey of expert opinion

**DOI:** 10.1186/s13017-015-0050-5

**Published:** 2015-11-19

**Authors:** Yoram Kluger, Ofir Ben Ishay, Massimo Sartelli, Amit Katz, Luca Ansaloni, Carlos Augusto Gomez, Walter Biffl, Fausto Catena, Gustavo P. Fraga, Salomone Di Saverio, Augustin Goran, Wagih Ghnnam, Jeffry Kashuk, Ari Leppäniemi, Sanjay Marwah, Ernest E. Moore, Miklosh Bala, Damien Massalou, Chirica Mircea, Luigi Bonavina

**Affiliations:** Rambam Health Care Center, Haifa, 31096 Israel; Department of Surgery, Macerata Hospital, Macerata, Italy; Papa Giovanni XXIII Hospital, Bergamo, Italy; Hospital Universitário Therezinha de Jesus, Faculdade de Ciências Médicas e da Saúde de Juiz de Fora (SUPREMA), Universidade Federal de Juiz de Fora (UFJF), Jiiz de Fora, Minas Gerais Brazil; Department of Surgery, University of Florida, Gainesville, Florida USA; Parma University Hospital, Parma, Italy; Faculdade de Ciências Médicas (FCM), Unicamp Campinas, Jiiz de Fora, Brazil; Ospedale Maggiore Carlo Albert, Bologna, Italy; Department of Surgery, University Hospital Center, Zagreb, Croatia; Mansoura Faculty of Medicine, Mansoura University Egypt, Mansoura, Egypt; Assia Medical Group, Assuta Medical Centers, Tel Aviv, Israel; Meilahti Hospital, Helsinki, Finland; Department of Surgery, Post-graduate Institute of Medical Sciences, Rohtak, India; Department of Surgery, Denver Health Medical Center, Denver, USA; Hadassah - Hebrew University Medical Center, Jerusalem, Israel; Hôpital St Roch 5, Université Nice Sophia-Antipolis, Nice, France; Department of General, Endocrine and Digestive Surgery, Saint-Louis Hospital, Université Paris, Paris, France; Policlinico San Donato, University of Milan School of Medicine, Milan, Italy

**Erratum to:***World Journal of Emergency Surgery* 2015, **10**:48 doi:10.1186/s13017-015-0043-4.

Following publication of the original version [1] of the article of the World Journal of Emergency Surgery 2015, it was bought to our attention that an authors name was incorrectly published. The authors name should be presented as “Augustin Goran” and not “Goran Augustin”.
